# Relationships between mental health and styles of help-seeking among care workers in comparison with the general population

**DOI:** 10.1007/s44192-025-00131-7

**Published:** 2025-02-06

**Authors:** Tomoko Matsui, Hikaru Oba, Ryosuke Uchida, Tsukasa Teraguchi

**Affiliations:** 1 Graduate School of Human Sciences, Osaka University, 1-2, Yamada-oka, Suita, Osaka Japan; 2 Graduate School of Health Sciences, Hirosaki University, Aomori, Japan; 3 Faculty of Human and Social Sciences, University of Marketing and Distribution Sciences, Hyogo, Japan; 4 Human Factor Research Group, Institute of Nuclear Safety System, Fukui, Japan

**Keywords:** Care workers, Nursing home, Help-seeking styles, Mental health, Dementia

## Abstract

**Background:**

Care workers in long-term nursing homes often experience burdens and a high rate of resignations. This study aimed to investigate the mental health of care workers and to examine the relationship between the tendency to seek help and their mental health, through a comparison with the general population to address this issue.

**Methods:**

Online surveys were conducted separately with 273 care workers and 260 general public using a questionnaire platform. They were asked about their demographic variables, mental health [the Kessler Psychological Distress Scale (K6) and stressors], help-seeking styles (self-directed, excessive, and avoidant help-seeking), and usage on use of psychological support services. The analyses were performed except for missing responses.

**Results:**

Overall, 132 (48.4%) care workers scored higher than the cutoff point on the K6. Regarding stressors identified by the care workers, most of the contents were related to work-related matters and workplace relationships, as demonstrated through qualitative analysis. Hierarchical multiple regression analysis showed that the interaction term between each help-seeking style and participant group was non-significant. Meanwhile, age (*β* = −0.116, *p* = 0.009), excessive help-seeking (*β* = 0.120, *p* = 0.014), and avoidant help-seeking (*β* = 0.331, *p* < 0.001) were associated with the K6.

**Conclusion:**

This study revealed the tendency of care workers’ help-seeking behavior and its relationship to mental health. Improving care workers’ help-seeking skills and adjusting their work environment are essential for maintaining and enhancing their mental health.

## Introduction

In Japan, care workers (CW) are mainly engaged in providing care for all aspects of the lives of older people with dementia in care facilities. Their duties include not only direct care (e.g., assistance with eating, toileting, and bathing), but also cleaning rooms, organizing events, communicating and coordinating with their family members and medical professionals, making work reports, and managing welfare equipment. Additionally, people can work as CW without specific qualifications. By completing the Care Worker Entry Training (equivalent to the former Home Helper Level 2 course), they can engage in tasks that require specialized knowledge and skills, such as physical care.

Although the turnover rate of care workers (CW) in Japan is declining, labor shortages have been a challenge for many years [1]. CW face a variety of issues, such as work overload; difficulties in relationships with users, their families, and colleagues; anxiety regarding changes in the system; and back pain [2–5]. CW showed strong stress reactions caused by events at work such as switching work schedules, working with people in other professions, and oral care [6]. Managing the behavioral and psychological symptoms of dementia is a stressful experience for them and is associated with various negative feelings, including care burden [7, 8]. Furthermore, the need for end-of-life care in nursing homes is expected to increase. A qualitative study of CW caring for the dying older adults showed that they were not fully accustomed to providing end-of-life care and experienced conflicts [9]. The increased burden on CW may lead to mental health issues, worsen workforce shortages, and even contribute to cases of abuse.

### The importance of maintaining care workers’ mental health

Research on CW’s mental health has increased. Burnout is a prolonged response to chronic emotional and interpersonal stressors at work [10] and has been used as an indicator of mental health among human service professionals. Most CW who work with long-term care residents with dementia experience low or moderate levels of burnout [11]. One study of home CW showed that conflicts with patients, their families, supervisors, and colleagues were positively associated with parts of burnout [12].

Few studies have used depression and anxiety as indicators. One study of staff members in long-term care facilities reported that 64.6% of them had poor mental health [13]. Mental health status during the COVID-19 pandemic was assessed among CW in a variety of settings (e.g., long-term healthcare facilities), and the results showed that more than half of them scored above the cut-off point for depression and anxiety [5, 14]. Factors related to mental health levels have been examined; for example, a good work environment (‘easy to discuss work concerns in their workspace’), a positive attitude toward end-of-life care, and generalized trust were positively associated with better mental health [13].

Many CW have mental health issues; however, a survey of CW in Japan reported that only 2.1% of them were vising a medical institution for mental illness [15]. One study in Canada reported that 13.2% of their samples who were long-term care staff and management had used mental health support or services during the COVID-19 pandemic [16]. Additionally, a study in Japan mentioned the most common reason for dissatisfaction with health and safety management was the inadequacy of measures taken for mental health; complaints about support from the institution included psychological stress, among CW in care facilities [15].

Existing research has suggested that many CW have poor mental health, and that measures to address this remain insufficient. The accumulation of knowledge on their mental health, including research on measures to address this issue, will be important.

### Mental health and help-seeking behavior

Help-seeking behavior (HSB) is defined as a problem-focused, planned behavior, involving interpersonal interaction with a selected healthcare professional [17], has been studied in various fields. Previous studies have examined factors and intervention methods related to healthcare utilization, consulting behaviors with others, and intentions to engage in these behaviors [18–22]. Studies have also focused on help-seeking styles [23–25]. It is defined by the characteristics of the process leading up to implementation and has been shown to generally predict actual behavior patterns, and is classified into three tendencies: ‘self-directed help-seeking (seeking help after trying to deal with the problem by oneself), excessive help-seeking (seeking help even when a problem is trivial), and avoidant help-seeking (not seeking help at all) [23, 24].’ People with an excessive help-seeking style reported higher social support, higher depression and sensitivity to rejection, and lower independence, while people with an avoidant help-seeking style reported higher independence, higher depression, and lower social support and self-determination, suggesting that they may have more support needs compared to the self-directed type [24].

There has been very little study on the characteristics of individuals’ help-seeking among CW. For instance, the relationship between help-seeking attitudes, burnout, and mental health was examined among social work certificate holders [26]. In a study of CW in group homes for older people with dementia, the relationship between avoidant action and cognitive stress coping and burnout [27].

While research on promoting the use of mental health services is certainly important, it is also important to conduct research that focuses on individual help-seeking styles that are directly related to their daily work. Understanding the relationship between individual HSB charactersitics and their mental health may help us consider more effective strategies that take into account the characteristics of the targets to promote their health. The objectives of this study were to (1) identify the mental health status of CW and (2) understand the relationship between the tendency to seek help and mental health among CW through a comparison with the general population (GP). The concepts of depression and burnout both overlap and differ, and no conclusions have been drawn regarding these identifications. Although many studies on CW have used burnout as an indicator of mental health, it is also important to understand their mental health challenges in the context of mental disorder, such as depression and anxiety, given subsequent consulting healthcare services and treatment.

## Materials and methods

### Procedure and participants

To reduce the burden on facility staff cooperating in the survey and the CW responding, and due to the difficulty of recruiting participants from the GP, we conducted two online surveys targeting CW and the GP between December 2021 and March 2022. The survey forms were developed using a questionnaire platform provided by Qualtrics, Ltd.

A survey of CW was conducted at the facilities of a social welfare corporation. The corporation’s contact personnel coordinated the survey, obtained cooperation from facility staff, and allowed participants access to the survey form website. Three hundred and fifteen care staff members expressed interest in participating in the survey. For public populations, we used the crowdsourcing services of CrowdWorks Ltd. The advertisement was posted on the CrowdWorks website and described the questionnaire as ‘an attitude survey of care experience and mental health.’ Interested members of CrowdWorks participated in the online survey. The recruitment limit was set at 500 and 413 responses were collected.

The Directed Question Scale (DQS) [28] was employed in both surveys to ensure the reliability of the responses. Seven hundred twenty-eight respondents confirmed the purpose of the survey, and 710 agreed to complete the survey and proceeded to answer. A total of 533 respondents were included in the analysis, excluding respondents who answered the DQS incorrectly (*n* = 110), respondents from the GP those who were engaged in medical and social work (*n* = 28), and respondents with a medical or welfare certification (*n* = 39).

### Measurements

#### Demographic variables

Participants were asked about age, sex, educational background, employment status, certifications (multiple responses), industrial category, and total years of service in nursing homes.

#### The Japanese version of Kessler Psychological Distress Scale (K6) [29–31]

We used the K6, which is widely used in Japan and consists of six items to screen psychological distress. This measured on a 5-point scale (0–4). The optimal cutoff point of the K6 for mood/anxiety disorders was estimated to be 4/5 [31]. Cronbach’s alpha coefficient was 0.91.

#### Contents of stressors

Respondents were asked to detail their stressors through an open-ended response.

#### Help seeking styles [23]

This scale consists of 12 items with three subscales (self-directed, excessive, and avoidant help-seeking) to assess help-seeking styles. Response options range from 1 (very unlikely) to 7 (very likely). In the present study, Cronbach’s alpha coefficients of each component were 0.88–0.94 (respectively, self-directed, excessive, and avoidant).

#### Information on the use of psychosocial support services

Participants were asked about their experience with using psychosocial support services to resolve their difficulties and problems (yes/no). Those who reported using such services were asked to select the services they used from the list in Table 1.

We asked the respondents about their status of and thoughts about their use of the services based on the transtheoretical model [32]. Those who had such experiences were asked to choose the one response from the followings: ‘Continued use for more than six months,’ ‘I have used such a service but not for more than six months,’ and ‘I had used bur currently not use.’

### Analysis

Descriptive statistics were calculated for each variable. For the univariate analysis, *t*-tests and correlation analyses were performed. Hierarchical multiple regression analysis was performed with scores of the K6 as the dependent variable, age and sex that correlated with in Step 1 (forced input), respective help-seeking style factors (each variable was converted to Z-scores) and groups (CW/GP) in Step 2 (forced input), and interaction terms between the respective help-seeking style factors (Z-score) and groups in Step 3 (forced input) to examine the relationship between help-seeking styles and mental health. Participants who provided incomplete answers were excluded from the analysis (pairwise). In each analysis, missing responses were excluded from the analysis. IBM SPSS Statistics version 28 for Mac was used. The data on the content of their stressors was categorized and the categories were named by the first author using MAXQDA Analytics Pro 2020. The co-authors checked these categories and revisions were made based on their feedback.

### Ethical considerations

This study was approved by the Ethics Committee of the Graduate School of Human Sciences (Behavioral Sciences) at Osaka University (reference number: HB021-081). The purpose of the survey was explained on the survey website. Participants were considered to have provided consent by responding to the survey.

## Results

### Participants (Table 1)

**Table 1:** Demographic information of the participants

	General public (*n* = 260)		Care workers (*n* = 273)	
	Mean (*n*)	SD (%)	Mean (*n*)	SD (%)
**Age**	41.1	9.9	43.4	12.3
**Sex**				
Male	107	41.2	102	37.4
Female	151	58.1	167	61.2
No response	2	0.8	4	1.5
**Highest education**				
Graduate School	19	7.3	3	1.1
University or vocational school	188	72.3	142	52.0
High school	48	18.5	108	39.6
Junior high school	2	0.8	12	4.4
Others	3	1.2	8	2.9
**Certification**				
Care worker	0	0	190	69.6
Home helper	0	0	97	35.5
Social worker	0	0	23	8.4
Care manager	0	0	7	2.6
Others	12	4.6	23	8.4
No certification	248	95.4	8	2.9
**Number of years of service**	–	–	8.8	5.4
**Employment status**				
Full-time employees	81	31.2	204	74.7
Others (part-timer workers, contract workers)	117	45.0	69	25.3
Unemployed	62	23.8	0	0
	(*n* = 226)		(*n* = 273)	
**Psychosocial support services use**				
Never used before	160	70.8	222	81.3
Have used	66	29.2	51	18.7
**Details**				
Currently using	19	8.4	8	2.9
Have used in the past	47	20.8	41	15.0
No response	0	0	2	0.7
**Details of psychosocial support services used by participants**				
Psychiatry	17	7.5	6	2.2
Psychosomatic medicine	42	18.6	26	9.5
Counseling by therapist	30	13.3	27	9.9
Telephone consultation	6	2.7	0	0
E-mail consultation	4	1.8	0	0
Others	0	0	1	0.4
No response	0	0	2	0.7

Of the 533 participants, 260 were from the GP and 273 were CW. The most common highest education level was university or vocational school in both group (GP: *n* = 188,72.3%); CW: *n* = 142, 52.0%). Most of the GP had no certifications (*n* = 248, 95.4%), while CW mostly had care worker certifications (*n* = 190, 69.6%).

### Mental health status, contents of stressors, and utilization of psychosocial support services

The *t*-test on the K6 scores showed that significantly higher for the GP [*t*(508) = 2.144, *p* = 0.033, *d* = 0.190]. Focusing on cutoff score, 138 (58.2%) members of the GP and 132 (48.4%) CW had scores of 5 or higher. The chi-square test showed a significant association between groups and cutoff scores (*χ*^2^ = 4.967, *df* = 1, *p* = 0.026), with significantly more of the GP scoring above the cutoff (*p* < 0.05).

The classification of stressors resulted in ten categories, and some categories included subcategories (Table 2). The most common responses for both groups were *work, study and housework* (27.8%), *interpersonal relationships* (23.5%). For the GP, including subcategories, most of the content was related to *work-related matters* (25.3%) *and financial issues* (20.3%). For CW, most of the contents were related to *work-related matters* (27.5%) and *workplace relationships* (14.7%).

**Table 2:** Contents of stressors

	All (*n* = 510)		General population (*n* = 237)		Care workers (*n* = 273)	
	*n*	%	*n*	*%*	*n*	*%*
**Work, study, and housework**	142	27.8	67	28.3	75	27.5
Work-related matters	135	26.5	60	25.3	75	27.5
Housework	9	1.8	7	3.0	2	0.7
Academic content	4	0.8	4	1.7	0	0
**Interpersonal relationships**	120	23.5	57	24.1	63	23.1
Workplace relationships	64	12.5	24	10.1	40	14.7
Family relationships	30	5.9	19	8.0	11	4.0
Relationships with partners/friends/acquaintances/neighbors	9	1.8	8	3.4	1	0.4
Others (relationships)	45	8.8	21	8.9	24	8.8
**Mental and physical health**	63	12.4	46	19.4	17	6.2
Physical health	33	6.5	24	10.1	9	3.3
Mental health	17	3.3	11	4.6	6	2.2
Others (mental and physical health)	17	3.3	15	6.3	2	0.7
**Financial issues**	59	11.6	48	20.3	11	4.0
**Family health, caregiving, childcare, and life events**	54	10.6	47	19.8	7	2.6
Family health issues/caregiving	26	5.1	24	10.1	2	0.7
Raising children	24	4.7	19	8.0	5	1.8
Life events	6	1.2	5	2.1	1	0.4
**COVID-19, disasters, environment**	27	5.3	22	9.3	5	1.8
Influences of COVID-19 and disasters	22	4.3	17	7.2	5	1.8
Environment	8	1.6	8	3.4	0	0
**Anxiety about one's future**	26	5.1	23	9.7	3	1.1
**Issues related to existence, roles, and self**	12	2.4	6	2.5	6	2.2
**Others**	28	5.5	10	4.2	18	6.6
**Nothing**	163	32.0	54	22.8	109	39.9

Twenty-three participants did not respond in the general population group

Regarding the use of psychosocial support services (Table 1), 66 (29.2%) of the GP (*n* = 226) and 51 (18.7%) of the CW (*n* = 273) have used some type of service. Both CW and the GP frequently used psychosomatic services.

### The relationship between the help-seeking tendency and mental health

We examined the association between demographic variables, the K6, and help-seeking style (Table 3). The results of the GP (*n* = 207–260) showed that significant associations between age and the K6 (*r* = −0.15), age and self-directed help-seeking (*r* = −0.13), sex and excessive help-seeking (*r* = 0.14), sex and avoidant help-seeking (*r* = −0.26), and the K6 and avoidant help-seeking (*r* = 0.24) (*ps* < 0.01–0.05). The results of the correlation analysis among CW (*n* = 271–273) showed that significant associations between age and the K6 (*r* = −0.15), sex and the K6 (*r* = −0.12), sex and self-directed help-seeking (*r* = −0.22), and the K6 and avoidant help-seeking (*r* = 0.31) (*ps* < 0.01–0.05).

**Table 3:** Correlation analysis of variables by groups

	Care workers						General population (*n* = 207–260)		Care workers (*n* = 271–273)	
General population	1	2	3	4	5	6	*M*	*SD*	*M*	*SD*
1 Age	–	0.26**	−0.15*	−0.09	−0.10	0.10	41.07	9.87	43.36	12.31
2 Sex (male = 1, female = 2)	−0.12*	–	−0.12*	0.01	−0.07	−0.22**	1.60	0.51	1.64	0.51
3 Total score of the K6	−0.15*	0.01	–	0.01	0.31**	0.00	6.63	5.64	5.59	5.31
4 Excessive help-seeking	0.00	0.14*	−0.01	–	−0.38**	−0.11	12.82	6.08	13.81	6.72
5 Avoidant help-seeking	−0.08	−0.26**	0.24**	−0.50**	–	0.22**	13.14	6.07	11.32	6.27
6 Self-directed help-seeking	−0.13*	0.04	−0.11	−0.25**	0.08	–	20.34	4.42	17.29	5.68

** p < 0.01, * p < 0.05

Upper row: results for care workers, lower row: results for the general population

Hierarchical multiple regression analysis showed that the interaction term between each help-seeking style and group was non-significant; therefore, Model 2 was adopted (Table 4). Age (*β* = −0.116, *p* = 0.009), excessive help-seeking (*β* = 0.120, *p* = 0.014), and avoidant help-seeking (*β* = 0.331,* p* < 0.001) were associated with the K6.

**Table 4:** The K6 associated with help-seeking styles

	Model 1				Model 2				Model 3			
	B	95.0% CI for B	*β*	*p*	B	95.0% CI for B	*β*	*p*	B	95.0% CI for B	*β*	*p*
Step 1												
Age	−0.073	−0.117 to −0.030	−0.150	0.001	−0.057	−0.099 to −0.014	−0.116	0.009	−0.057	−0.100 to −0.015	−0.118	0.008
Sex (male = 1, female = 2)	−0.497	−1.458 to 0.465	−0.046	0.310	−0.142	−1.073 to 0.789	−0.013	0.765	−0.128	−1.074 to 0.819	−0.012	0.791
Step 2												
Excessive help-seeking					0.656	0.135 to 1.177	0.120	0.014	0.619	−0.040 to 1.279	0.113	0.065
Avoidant help-seeking					1.812	1.283 to 2.341	0.331	<0.001	1.898	1.203 to 2.592	0.347	<0.001
Self-directed help-seeking					−0.498	−0.996 to 0.000	−0.091	0.050	−0.378	−0.998 to 0.241	−0.069	0.231
Group (CW = 0, GP = 1)					0.756	−0.218 to 1.730	0.069	0.128	0.813	−0.173 to 1.799	0.074	0.106
Step 3												
Excessive help-seeking × Group									0.016	−1.071 to 1.104	0.002	0.977
Avoidant help-seeking × Group									−0.238	−1.312 to 0.836	−0.029	0.663
Self-directed help-seeking × Group									−0.388	−1.455 to 0.680	−0.041	0.476
*R*^2^				0.026			0.120				0.122	
Δ*R*^2^							0.094	(*p* < 0.001)			0.002	(*p* = 0.825)

## Discussion

This study aimed to understand the present situation regarding the mental health of CW and clarify the relationship between an individual help-seeking tendencies and mental health among CW, through comparisons with the GP.

### Mental health status and use of services

The results showed that, compared with the GP, CW had better mental health and a lower proportion of those who scored above the cutoff point on the K6. However, around half of the CW had a K6 score of 5 or higher, suggesting that they might have mood/anxiety disorders. This is similar to or lower than the findings of previous studies, although the scales used for measurement differ [5, 13, 14]. Additionally, in the Comprehensive Survey of Living Conditions [33], the proportion of those who scored 5 points or higher was 20–30% among participants aged 20 or older. Regarding stressors, the responses of CW were mostly related to work-related matters and relationships in the workplace, similar to previous studies [2–5]; not many responses were given for other items. The previous study pointed to interpersonal relationships in the workplace as a risk factor for CW’s mental health [5], and this study support it in the qualitative aspects, although the results of this study include interpersonal relationships outside of work. On the other hand, the GP responded more frequently to items related to finances, support for others, mental and physical health, and concerns about the future, in addition to work and interpersonal problems.

Regarding the use of psychosocial support services, 2.9% of CW were currently using them, which is similar to previous studies [15], and it was less than the general population of this study. According to one study [34], CW showed that they asked help earlier than the GP for problems related to BPSD in older adults. Support for CW to use such services for their own care might also be important.

### Relationship between help-seeking style and mental health among CW

We found that an avoidant help-seeking style and excessive help-seeking style were positively related to the K6 scores among both CW and the GP. This finding supports previous study [24].

People with an excessive help-seeking style seek help when they have a problem, often even if the problem is trivial. They reported higher social support, also higher depression and sensitivity to rejection, and lower independence, suggesting that they may have support needs compared to the self-directed type [24]. Given these characteristics, it is necessary to understand the background of those with a higher level of excessive help-seeking. Furthermore, for those who help people with such a help-seeking style, it may be burdensome to be asked too many questions or consulted more than necessary.

Participants with a higher level of avoidant help-seeking were shown to have a stronger association with the K6 scores than those with other styles. They reported higher independence and depression and lower social support and self-determination, suggesting that they have higher support needs compared with the self-directed type [24]. The previous study reported that avoidant action and cognitive stress coping was positively associated with emotional exhaustion and depersonalization components of burnout among CW in group homes for people with dementia [27]. In the field of older adult care, CW are not only required to consult with and report to their colleagues and managers but they are sometimes also required to work in collaboration with other professionals. Thus, not seeking help when they have a problem will not only worsen their problem, but may also cause other problems on several fronts. It may not only increase the workers suffering but may also cause disadvantages for service users and their families.

The results of this study suggest that higher levels of excessive and avoidant help-seeking were associated with poorer mental health, which is consistent with previous findings. In addition to mental health, one study [35], not in the context of CW, examined the relationship between job-related HSB and performance ratings, and showed that the relationship was curvilinear. One study [35] examined the relationship between job-related HSB and performance ratings, and showed that the relationship was curvilinear; however, this finding was not in the context of CW. This suggests that seeking either a lot of help or less help has a negative impact on job performance. Thus, it is necessary to understand the underlying factors among those who have an excessive/avoidance help-seeking style and provide educational opportunities or systems to enhance help-seeking skills.

While interpersonal relationships are an essential component of help-seeking behavior [17], they can also be a source of stress for CW. Establishing an environment in which consultations and reports can be made more easily is needed while considering a comfortable relationship for both the providers and recipients of help. A study showed that a good work environment, positive attitude toward end-of-life care, and generalized trust were positively associated with better mental health [13]. Moreover, efforts to reduce workload and appropriate evaluation and staffing can reduce turnover intentions, mediated by efforts to improve the quality of care [36]. Consequently, when staffing, for example, considering the balance of assigning people with excessive help-seeking and those with avoidant help-seeking to an environment where they are more likely to seek help can directly or indirectly contribute to improved mental health and reduced turnover intentions. For example, from a health management perspective, it is important not only to conduct regular stress assessments but also to create an environment where psychological counselors or occupational physicians can provide support to staff exhibiting such tendencies in the workplace. Additionally, staff should also be encouraged to utilize external psychosocial support services when necessary.

### Limitations and future research

The survey of the GP was a web-based survey, which may have resulted in selection bias. In the survey of CW, those unaccustomed to answering online questionnaires may have avoided responding. Additionally, regarding CW, the survey was conducted at facilities run by a specific company, which may have led to biased responses. Considering the above, it is difficult to generalize the study results. In future studies, participants should be recruited in the same setting as far as possible. Furthermore, CW and the GP selected for this survey differ in elements such as utilization history of psychosocial support services. Therefore, further consideration may be needed in the future to explore the basis of these differences between CW and GP. In addition, as this survey was conducted during the COVID-19 pandemic, changes in work habits and lifestyles may have affected the results. Despite the limitations, this study has provided valuable insights that contribute to improving the mental health of CW.

## Conclusion

This study revealed the current status of the mental health of care workers and the relationship between mental health and help-seeking behavior, through comparison with the general population. Although maximizing rewards is also necessary to reduce turnover and to respond to the diversity of individual needs [37], a study found that while reward is important for improving the quality of life of care workers, it is not as important as before when compared to other factors such as relationships [15]. The findings of this study may be useful in developing support strategies and training methods for care workers based on their individual characteristics, as well as in considering the content of their work, including staffing and other aspects of their job. We expect that it will be utilized in the education and training of care workers and in providing them support in the workplace, and that measures will be taken to maintain and improve the mental health of care workers.

## Data Availability

The datasets used in this study are not openly available because it contains data which will be used in the future. They are available from the corresponding author upon reasonable request.
